# Demanding New Honey Qualitative Standard Based on Antibacterial Activity

**DOI:** 10.3390/foods9091263

**Published:** 2020-09-09

**Authors:** Marcela Bucekova, Veronika Bugarova, Jana Godocikova, Juraj Majtan

**Affiliations:** Laboratory of Apidology and Apitherapy, Department of Genetics, Institute of Molecular Biology, Slovak Academy of Sciences, Dubravska cesta 21, 845 51 Bratislava, Slovakia; marcela.bucekova@gmail.com (M.B.); veronika.bugarova@savba.sk (V.B.); jana.godocikova@savba.sk (J.G.)

**Keywords:** hydrogen peroxide, functional food, bacterial pathogen, commercial honey, quality standard

## Abstract

Honey is a functional food with health-beneficial properties and it is already used as a medical device in wound care management. Whether ingested orally or applied topically, honey must fulfill the requirements of international standards based on physicochemical characteristics. However, there is an urgent need for some additional standards reflecting biological properties. The aim of the study was to evaluate the antibacterial activity of 36 commercial honey samples purchased from supermarkets and local food shops and compare their efficacy to that of three honey samples from local beekeepers and three types of medical-grade honey. Furthermore, the hydrogen peroxide (H_2_O_2_) content and protein profile were assessed in all honey samples. Analysis of the antibacterial activity of commercial honeys revealed that 44% of tested samples exhibited low antibacterial activity, identical to the activity of artificial honey (sugars only). There was a significant correlation between the overall antibacterial activity and H_2_O_2_ content of honey samples. However, in some cases, honey samples exhibited high antibacterial activity while generating low levels of H_2_O_2_ and vice versa. Honey samples from local beekeepers showed superior antibacterial activity compared to medical-grade honeys. The antibacterial activity of honey can be easily altered by adulteration, thermal treatment or prolonged storage, and therefore it fulfils strict criteria to be suitable new additional quality standard.

## 1. Introduction

Honey, a traditional sweetener, is considered as a functional food, and several recent clinical studies have proved its health-beneficial properties such as improving lipid profile [1], reducing postoperative pain [2] and inflammation [3] and modulating of hypertension [4]. Besides its oral consumption, honey has successfully been used topically in the treatment of a broad spectrum of surgical and chronic wounds [5,6] and mucositis [7] as well as herpes simplex labialis [8]. Whether applied topically or ingested orally, honey must fulfil all the requirements of international standards and possesses proven biological activity. In addition, honey has to be sterilised by gamma radiation when used in wound care management. In the case of medical usage, only honey of high quality and with guaranteed biological activity should be a part of honey-based medical products. 

Several quality standards have been recongnised and listed as ‘Current international honey standards’ which are specified in the European Honey Directive (2002) [9] as well as in the Codex Alimentarius Standard for Honey (2001) [10]. Honey samples meeting all strictly defined composition criteria, including mainly moisture, sucrose and hydroxymethylfurfural (HMF) content, are recommended for human consumption and can be placed on the market. 

On the other hand, the range of particular current honey standards is too wide and, in most cases, adulterated and/or heat-processed honey is still within the range for tested criteria and therefore classified as honey with proved quality. Furthermore, most importantly, none of the above-mentioned criteria are related to the biological activity of honey.

One of the most important and well-described aspects of honey’s biological activity is its antibacterial activity that is mediated via multiple mechanisms of action such as osmotic pressure, low pH value and water activity, and disruption of bacterial cell membranes due to the presence of antibacterial peptide defensin-1.

Bee defensin-1, a regular but quantitatively variable antibacterial component of honey [11], is mainly effective against Gram-positive bacteria [12,13,14]. Furthermore, defensin-1 possesses antibiofilm activity against established multi-species biofilm [15] and exhibits wound healing properties [16].

After dilution, the antibacterial activity of honey is mainly mediated via enzymatically generated hydrogen peroxide (H_2_O_2_) in the diluted honey [17], excluding manuka honey where H_2_O_2_ is not accumulated [18]. This activity can be negatively affected by uncontrolled thermal processing or prolonged storage [19]. The major antibacterial compound in manuka honey is methylglyoxal (MGO) [20,21] that is primarily able to inhibit the growth of Gram-positive bacteria [22,23]. On the other hand, MGO in manuka honey seems to be ineffective against *Pseudomonas aeruginosa* [24]. Similar to that of different blossom honeys, the total antibacterial action of manuka honey is based on multiple mechanisms of action rather than single components. Therefore, the antibacterial potential of honey could be a suitable new additional international quality standard.

Our recent study aimed to determine the antibacterial efficacy of 233 different blossom honey samples collected in Slovakia [25]. Linden honeys showed the greatest antibacterial efficacy followed by sunflower, multi-floral and acacia honey samples. The lowest antibacterial activity was assessed in rapeseed honey samples.

The goal of the study was to (i) evaluate the antibacterial potential of commercial honeys purchased from supermarkets (*n* = 19) and local shops (*n* = 17) in Slovakia, (ii) characterise the protein profile of honey samples and (iii) determine the overall H_2_O_2_ content in honey samples. Moreover, antibacterial activity together with the capability to generate H_2_O_2_ was assessed in three honey samples from local beekeepers and in three medical-grade honey samples in order to compare the overall antibacterial potential of different honeys.

## 2. Materials and Methods

### 2.1. Honey Samples

A total of 36 commercial samples of honey purchased from supermarkets in 2017 (*n* = 19) or local food shops in 2019 (*n* = 17) were evaluated. Most of the samples, particularly from those from local food shops, declared the botanical and geographical origin on the label (Table 1). 

Three honey samples (acacia, linden and honeydew) from local beekeepers and three different commercial products based on medical-grade honeys: Vivamel^®^ (Toasama, Domzale, Slovenia), Revamil^®^ (Bfactory, Rhenen, Netherlands) and Activon Tube^®^ (Advancis Medical, Nottingham, UK) were also evaluated. All samples were immediately stored at 4 °C in the dark.

As a negative control artificial honey was prepared by dissolving 39 g d-fructose, 31 g d-glucose, 8 g maltose, 3 g sucrose, and 19 g distilled water, as described elsewhere [26].

### 2.2. Microorganisms

The tested bacterial isolates *Pseudomonas aeruginosa* CCM1960 and *Staphylococcus aureus* CCM4223 were acquired from the Department of Medical Microbiology at the Slovak Medical University in Bratislava, Slovakia.

### 2.3. Determining the Antibacterial Activity 

The honey samples were subjected to an antibacterial minimum inhibitory concentration (MIC) assay to determine the antibacterial activity against *P. aeruginosa* and *S. aureus* following the method of Bucekova et al. [27]. Bacteria were cultured in Mueller-Hinton broth (MHB) at 37 °C overnight. Bacterial culture was suspended in phosphate-buffered saline (PBS), with a pH of 7.2, and the turbidity of the suspension was adjusted to 10^8^ colony-forming units (CFU)/mL and diluted with MHB medium (pH 7.3 ± 0.1) to a final concentration of 10^6^ CFU/mL. The final volume in each well of sterile 96-well polystyrene U-shaped plates (Sarstedt, Germany) was 100 μL, consisting of 90 μL of sterile MHB medium or diluted honey sample and 10 μL of bacterial suspension. After 18 h incubation at 37 °C and 1250 rpm, the inhibition of bacterial growth was determined by monitoring the optical density at 490 nm using a Synergy HT microplate reader (BioTek Instruments, Winooski, VT, USA). The final MIC values correspond to the lowest concentrations of honey that completely inhibited bacterial growth. All the tests were performed in triplicate and repeated three times. 

Each honey sample dilution was prepared from a 50% honey solution (*w*/*w* in MHB medium) by further dilution with the MHB medium, resulting in final concentrations of 40%, 35%, 30%, 25%, 20%, 18%, 16%, 14%, 12%, 10%, 8%, 6% and 4%.

### 2.4. Determining the H_2_O_2_ Content

The H_2_O_2_ content in the honey samples was determined using a Megazyme GOX assay kit (Megazyme International Ireland Ltd., Bray, Ireland) based on the release of H_2_O_2_ after glucose oxidase catalysis of the oxidation of β-d-glucose to d-glucono-δ-lactone. As a standard, 9.8–312.5 μM diluted H_2_O_2_ was used. Honey solutions (40% *w*/*w* in 0.1 M potassium phosphate buffer, pH 7.0) were prepared and immediately measured. Each honey sample and H_2_O_2_ standard was tested in duplicate in a 96-well microplate. The absorbance of the reaction was measured at 510 nm using a Synergy HT microplate reader (BioTek Instruments, Winooski, VT, USA).

### 2.5. Determining the Protein Profile of Honey Samples

For protein determination, 15 μL aliquots of diluted honey samples (50% *w*/*w* in distilled water) were loaded on 12% SDS-PAGE gels and separated using a Mini-Protean II electrophoresis cell (Bio-Rad, Hercules, CA, USA). Protein content was assessed after gel staining with Coomassie Brilliant Blue R-250 (Sigma-Aldrich, Darmstadt, Germany) or Serva Blue (Serva, Heidelberg, Germany).

### 2.6. Statistical Analysis

The Pearson correlation test was used to analyse the correlation between the antibacterial activity and the H_2_O_2_ content in the honey. The data are expressed as mean values with the standard deviation (SD). Data with *p* values smaller than 0.05 were considered statistically significant. GraphPad Prism was used to perform all the statistical analyses (GraphPad Software Inc., La Jolla, CA, USA).

## 3. Results

### 3.1. Antibacterial Activity of Commercial Honey Samples

**Figure 1:**
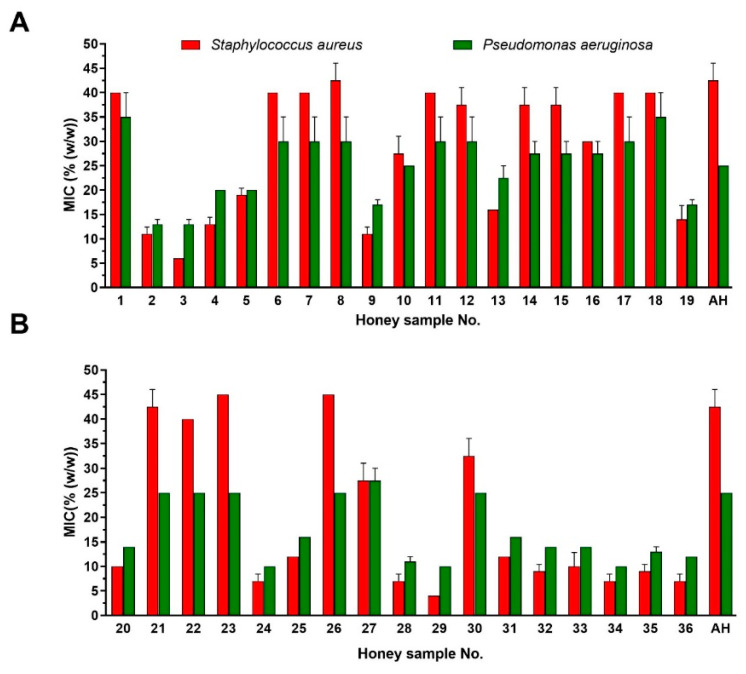
Antibacterial activity of the commercial honey samples (*n* = 36) purchased in Slovakia. The antibacterial activity was evaluated against two bacterial pathogens by a minimum inhibitory concentration (MIC) assay. (**A**) Honey samples purchased from supermarket (*n* = 19) (**B**) Honey samples purchased from local food shop (*n* = 17). The data are expressed as the mean values with the standard deviation (SD). AH—artificial honey.

Antibacterial activity was determined in commercial (*n* = 36), local beekeeper (*n* = 3) and medical-grade (*n* = 3) honey samples as well as in artificial honey. The activity of the commercial honey samples against bacteria was expressed as an MIC value (Figure 1). Commercial honey samples from local food shops (Nos. 20–36) exhibited a greater antibacterial effect compared to samples from supermarkets (Nos. 1–19). The MIC values of about 50% of supermarket honey samples against *S. aureus* were identical to those of artificial honey, compared to 25% of honey samples from local food shops. The average MIC value of supermarket and local food shop honey samples against *S. aureus* was 28.6% and 19.2%, respectively. The highest antibacterial activity was exhibited by honey sample No. 29 with an MIC value of 4% against *S. aureus*.

Artificial honey at a concentration of 25% was able to inhibit the bacterial growth of *P. aeruginosa*. Due to the high susceptibility of *P. aeruginosa* to the sugar content in honey samples, this bacterium is not a suitable model for testing the antibacterial activity of honey.

### 3.2. H_2_O_2_ Content of Commercial Honey Samples

**Figure 2:**
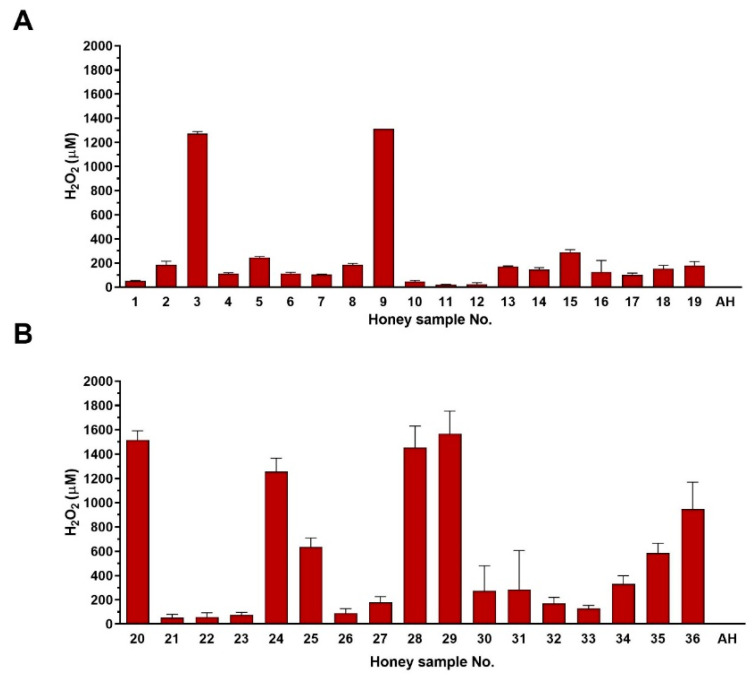
Hydrogen peroxide (H_2_O_2_) content in commercial honey samples (*n* = 36). (**A**) supermarket honey samples (*n* = 19), (**B**) local food shop honey samples (*n* = 17). Determination of the H_2_O_2_ content was carried out in diluted honey samples (40% (*w*/*w*) after 24 h of incubation at 37 °C. The data are expressed as the mean values with the standard deviation (SD). AH—artificial honey.

H_2_O_2_, a major antibacterial compound in honey, was determined in 36 commercial honey samples (Figure 2). The average H_2_O_2_ content in supermarket and local food shop honey samples was 256 and 565 µM, respectively. Honey samples purchased in local food shops were more potent in H_2_O_2_ production (Figure 2B). Three samples from supermarkets (sample Nos. 10, 11 and 12) accumulated very low levels of H_2_O_2_, at concentration below 50 µM (Figure 2A). 

**Figure 3:**
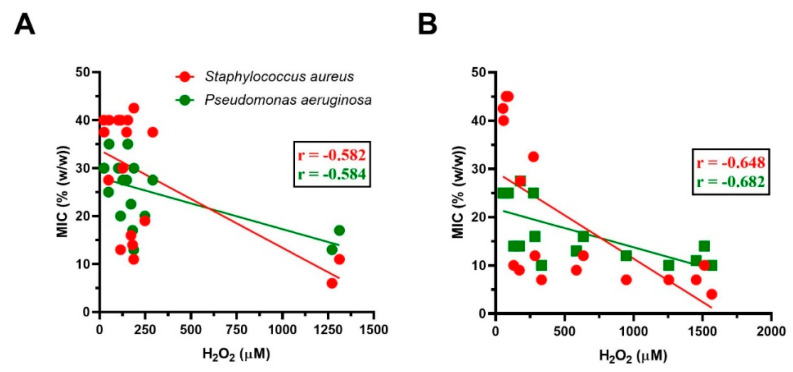
The relationship between the content of H_2_O_2_ and the antibacterial activity of commercial honeys: (**A**) supermarket honey samples (*n* = 19), (**B**) local food shop honey samples (*n* = 17) against two bacterial isolates. A Pearson correlation test was used for the correlation analysis.

The correlation analysis revealed a significant correlation between the H_2_O_2_ values and the antibacterial activity of both supermarket and local shop honey samples against *S. aureus* (*r* = −0.582, *p* < 0.01; *r* = −0.648, *p* < 0.01) as well as *P. aeruginosa* (*r* = −0.584, *p* < 0.001; *r* = −0.682, *p* < 0.01) (Figure 3).

Despite a significant correlation between the content of H_2_O_2_ and the antibacterial activity of all commercial honeys was revealed, some of tested honey samples (for example Nos. 19 and 33) did not show any correlation among MICs and H_2_O_2_ content.

### 3.3. Antibacterial Activity and H_2_O_2_ Content of Local and Medical-Grade Honey Samples

**Figure 4:**
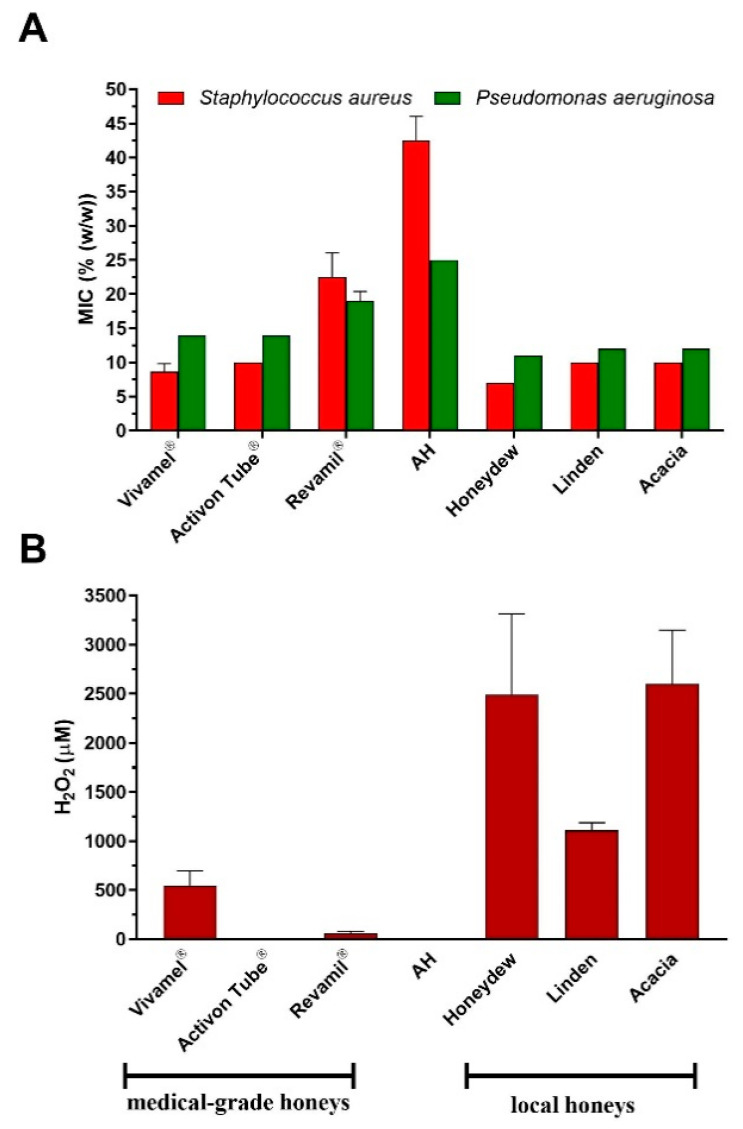
Antibacterial activity and the content of H_2_O_2_ in the honey samples from local beekeepers (*n* = 3) and medical-grade honey (*n* = 3). (**A**) The antibacterial activity was evaluated against two bacterial pathogens by a minimum inhibitory concentration (MIC) assay. (**B**) Determination of the H_2_O_2_ content was carried out in diluted honey samples (40% (*w*/*w*) after 24 h of incubation at 37 °C. The data are expressed as mean values with the standard deviation (SD).

Samples of three different types of honey (honeydew, linden and acacia) from local beekeepers and three medical-grade honeys were used for the determination of antibacterial activity. The antibacterial activity of these samples is shown in Figure 4A. All honeys from local beekeepers exhibited high antibacterial activity, with average MIC values ranging from 7% to 12% depending on the bacterium. The antibacterial effect of local honey samples was even higher than that of medical-grade honeys where MIC values were in the range from 9% to 22.5%. Among the medical-grade honeys tested, the lowest antibacterial activity was documented for Revamil^®^. The local honeys, honeydew and acacia honey samples in particular, produced a high level of H_2_O_2_ (Figure 4B). Interestingly, the level was significantly higher than in medical-grade honeys. Activon Tube^®^, a manuka honey based-wound care product, did not accumulate H_2_O_2_ due to inactivated glucose oxidase enzyme. Revamil^®^, a blossom honey-based product, produced a low level of H_2_O_2_ and thus showed moderate antibacterial activity. 

## 4. Discussion

Natural functional foods including honey with diverse biological activity have become very popular among consumers and a plethora of studies have discovered a number of bioactive ingredients that can provide a beneficial effect on human health. However, rising demand for honey and natural healthy foods in general causes the limited availability and high price of honey and thus honey has become the object of adulteration. International honey standards need to be updated since nowadays more adulterated honeys meet currently defined limits in term of quality. Furthermore, industrial processing of honey such as liquefaction or thermal treatment causes an increase in HMF, the major indicator of honey freshness and overheating during processing, but to values below the maximum permissible level of 40 mg/kg of honey [28,29,30,31]. Similarly, another standard, diastase activity (DN), also changes after thermal processing but, in most cases, the minimum permissible level of 8 is maintained [29,32]. As mentioned before, international honey standards do not take into account the biological effects of honey, although some of the effects including the antibacterial effect are well described and have proved to be sensitive parameters. Antibacterial activity is also the sole criterion for selecting honey for medical usage. 

In this study, we demonstrated that the antibacterial activity of commercial honeys purchased in supermarkets and local food shops is not uniform. Unfortunately, the antibacterial activity of more than 40% of the tested commercial honeys was identical to that of artificial honey. According to protein profile analysis of all honey samples, only three samples (Nos. 10, 11 and 12) showed a very low protein content (Figure S1). Missing crucial protein components, including bee-derived glucose oxidase, in these three samples resulted in negligible accumulation of H_2_O_2_. The typical protein profile was recognised in the rest of the honey samples, showing major royal jelly protein 1 (MRJP1) as the dominant honey protein (Figures S1 and S2) with molecular weight of 55 kDa.

The MRJP1 protein is multifunctional and has a nutritional function in larval jelly [33] and a presumed function in the bee brain that is associated with learning ability [34]. It also acts as a precursor protein of short antimicrobial peptide jelleines [35], however, its direct antibacterial activity is questioned [36,37].

Based on present and previous results [17], H_2_O_2_ content is not a suitable parameter for determining honey quality, even though a statistically significant correlation between antibacterial activity and H_2_O_2_ content was calculated. In some cases, honey samples exhibited high antibacterial activity while generating low levels of H_2_O_2_ and vice versa. Similarly, analysis of Polish honey samples revealed that some samples with the highest antibacterial activity were characterised by low levels of H_2_O_2_ [38]. In addition, we have recently showed that linden Slovak honey samples, which exhibited the strongest antibacterial effect, showed weak or no correlation between the antibacterial activity and H_2_O_2_ content [25]. Therefore, we assume that some certain types of honey (e.g., linden honey) may contain either non-peroxide antibacterial factors or specific minor components which act synergistically with other components to increase honey antibacterial potential.

Therefore, the antibacterial activity of honey depends on the presence of H_2_O_2_ but its concentration can vary from honey to honey and the presence of some additional minor constituents such as polyphenols is essential for a pronounced antibacterial effect. Honey as a highly complex product contains various known and yet unknown compounds of botanic origin that might alter H_2_O_2_ levels. In addition, the interaction of H_2_O_2_ with some particular polyphenols and/or other minor constituents can result in augmentation of honey antibacterial activity [17]. A very recent study by Brudzinski (2020) discussed the role of the colloidal structure of honey in H_2_O_2_ production, suggesting a relationship between the concentration of macromolecules, the propensity to form colloidal assemblies, H_2_O_2_ production and antibacterial effect [39].

In this study, three different types of medical-grade honey-based wound care products were evaluated. All products consist of 100% honey and no other additives were added. In contrast to natural honey, medical-grade honey undergoes a process of sterilisation, usually by gamma radiation. Gamma radiation of honey eliminates vegetative microbial cells as well as microbial spores [40,41,42] without affecting the overall antibacterial activity of honey [40,42,43,44,45]. However, it is likely that the whole process of medical-grade honey manufacture, including tubing, packing, sterilising and storing processes, results in a loss of some antibacterial activity. Taking into account the entire manufacturing process, it is necessary to monitor the antibacterial activity of processed honey at each manufacturing step. 

In the case of manuka honey, the Unique Manuka Factor (UMF) grading system has been established, reflecting the concentration of MGO, a major antibacterial compound found in manuka honey. A very recent study [46] demonstrated that UMF grade, as an indicator of the antibacterial potential of manuka honey, surprisingly, does not correlate with the antibacterial efficacy of manuka honey, supposedly due to changes in MGO content over time. Therefore, the UMF grading system seems to be unreliable and there is a need to develop a more suitable system. Furthermore, UMF grading is solely based on the agar well diffusion assay using *S. aureus* as a model bacterium [47]. Nowadays, this is considered to have relatively low sensitivity and the obtained results need to be confirmed/updated by a more sensitive broth microdilution assay, a preferred method for determining the antibacterial effect of complex natural products. Lastly, a few recent studies have raised the issue of the dominant role of MGO in the antibacterial effect of manuka honey [24,46]. 

Last but not least, determination of honey antibacterial activity could give beekeepers a competitive advantage due to the high demand for biologically active honey. Detrimental changes in the antibacterial effect of honey induced by prolonged storage or uncontrolled heating at high temperature may also negatively affect other biological effects of honey such as antioxidant, anti-inflammatory and wound healing activity. Many of these types of activity are mediated by polyphenolic compounds which are a light/heat sensitive group of biologically active compounds [48]. Therefore, it is likely that honey with low antibacterial activity, identical to artificial honey, loses most of its health-beneficial properties and acts solely as a sweetener.

## 5. Conclusions

In conclusion, analysis of the antibacterial activity of commercial honeys purchased in Slovakia revealed that more than 40% of the samples tested exhibited low antibacterial activity, identical to that of artificial honey (sugars only). There was a significant correlation between the overall antibacterial activity and H_2_O_2_ content of honey samples. However, in some cases, honey samples exhibited high antibacterial activity while generating low levels of H_2_O_2_ and vice versa. Honey samples from local beekeepers showed superior antibacterial activity compared to medical-grade honeys which are recommended for the treatment of infected wounds. The antibacterial activity of honey, as one of its well-described biological properties, should be used as an additional quality standard reflecting its biological properties. Further studies are needed to characterise the effect of long-term storage as well as heat processing on antibacterial activity of honey in order to improve this new potential quality standard.

## Figures and Tables

**Table 1:** Commercial honey samples tested in the study.

	No. of Honey Samples	Type of Honey	Geographical Origin of Honey
Supermarkets	1	multi-floral	Unknown *
	2	acacia	Slovakia
	3	honeydew	Slovakia
	4	linden	Unknown *
	5	multi-floral	Unknown *
	6	honeydew	Unknown *
	7	multi-floral	Unknown *
	8	multi-floral	Unknown *
	9	forest	Unknown *
	10	linden	Unknown *
	11	multi-floral	Unknown *
	12	acacia	Unknown *
	13	forest	Unknown *
	14	multi-floral	Unknown *
	15	multi-floral	Unknown *
	16	rapeseed	Slovakia
	17	multi-floral	Slovakia
	18	acacia	Slovakia
	19	honeydew	Slovakia
Local food shops	20	acacia	Slovakia
	21	multi-floral	Slovakia
	22	forest	Slovakia
	23	linden	Slovakia
	24	honeydew	Slovakia
	25	rapeseed	Slovakia
	26	multi-floral	Slovakia
	27	acacia	Slovakia
	28	honeydew	Slovakia
	29	honeydew	Slovakia
	30	multi-floral	Slovakia
	31	acacia	Slovakia
	32	multi-floral	Slovakia
	33	linden	Slovakia
	34	multi-floral	Slovakia
	35	multi-floral	Slovakia
	36	multi-floral	Slovakia

* honey originated from European union/non-European union countries.

## Supplementary Materials

The following are available online at https://www.mdpi.com/2304-8158/9/9/1263/s1, Figure S1: Protein profile of commercial honey samples (*n* = 19) from supermarkets and three samples from local beekeepers, Figure S2: Protein profile of commercial honey samples (*n* = 17) from local food shops.
