# Supplementary material for: Demanding New Honey Qualitative Standard Based on Antibacterial Activity
Source: Foods. 2020 Sep 9;9(9):1263. doi: 10.3390/foods9091263 (PMC7554693; doi:10.3390/foods9091263)
Supplement: Supplementary file 1 [file foods-09-01263-s001.pdf]

Supporting information

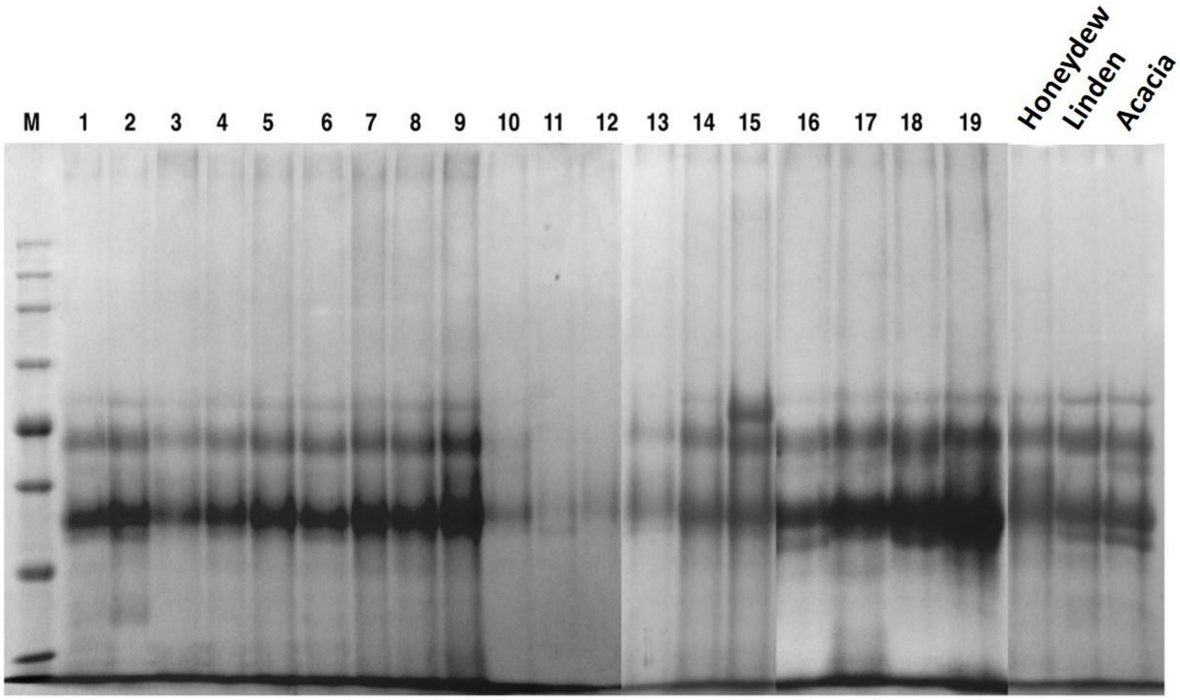

Figure S1. Protein profile of commercial honey samples (n=19) from supermarkets and three samples from local beekeepers. A 15 µL of diluted honey samples (50% *w*/*w* in distilled water) were separated by 12% SDS-PAGE gels and protein content assessed after gel staining with Coomassie Brilliant Blue R-250

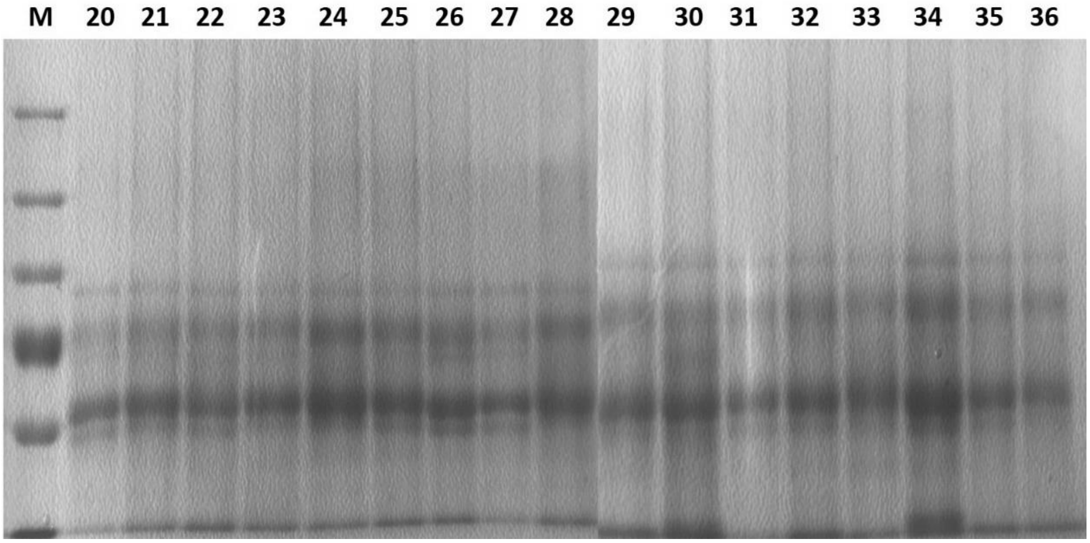

Figure S2. Protein profile of commercial honey samples (n=17) from local food shops. A 15 µL of diluted honey samples (50% *w*/*w* in distilled water) were separated by 12% SDS-PAGE gels and protein content assessed after gel staining with Serva blue.
